# The Evolution of Legislation in the Field of Medically Assisted Reproduction and Embryo Stem Cell Research in European Union Members

**DOI:** 10.1155/2014/307160

**Published:** 2014-07-24

**Authors:** Francesco Paolo Busardò, Matteo Gulino, Simona Napoletano, Simona Zaami, Paola Frati

**Affiliations:** ^1^Department of Anatomical, Histological, Forensic and Orthopaedic Sciences, Sapienza University of Rome, Viale Regina Elena 336, 00161 Rome, Italy; ^2^Neuromed, Istituto Mediterraneo Neurologico (IRCCS), Via Atinense 18, Pozzilli, 86077 Isernia, Italy

## Abstract

Medically Assisted Reproduction (MAR), involving in vitro fertilisation (IVF), and research on embryos have created expectation to many people affected by infertility; at the same time it has generated a surplus of laws and ethical and social debates. Undoubtedly, MAR represents a rather new medical field and constant developments in medicine and new opportunities continue to defy the attempt to respond to those questions. In this paper, the authors reviewed the current legislation in the 28 EU member states trying to evaluate the different legislation paths adopted over the last 15 years and highlighting those EU countries with no specific legislation in place and MAR is covered by a general health Law and those countries in which there are no laws in this field but only “guidelines.” The second aim of this work has been to compare MAR legislation and embryo research in EU countries, which derive from different origins ranging from an extremely prohibitive approach versus a liberal one, going through a cautious regulatory approach.

## 1. Introduction

Although medically assisted reproduction (MAR), involving in vitro fertilisation (IVF), has created expectation to many people affected by infertility, it has generated a surplus of laws and ethical and social debates. Undoubtedly, MAR represents a rather new medical field and constant developments in medicine and new opportunities continue to defy the attempt to respond to those questions [1]. However, problems involving MAR are even more extensive than what it may appear and only a few other areas of medicine have created many social and ethical questions and have drawn so much public interest as MAR. Basically, MAR tests the society, “not least because human reproduction is the means by which each society perpetuates itself and its traditions” [2], but mostly because the society is involved at all its levels: cultural, religious, social, medical, and legislative with different positions and points of view; MAR and human embryo research (HER) require more than any other medical fields the support and contribution of the entire society from legislators and physicians to human rights organizations and women's representatives [2].

The first aim of this paper is to review the current legislation in the 28 EU member states trying to evaluate the different legislation paths adopted over the last 15 years. The authors, at the same time, highlighted those EU countries with no specific legislation in place and MAR is covered by a general health law and those countries in which there are no laws in this field but only “guidelines,” which do not fully analyse this important field of medicine, with social, ethical, and legal issues.

The second aim of this work is to compare MAR and human embryo research legislation in EU countries, which have resulted sometimes in laws that curb access to certain procedures. These laws derive from different origins ranging from an extremely prohibitive approach versus a liberal one, going through a cautious regulatory approach. The authors aim to focus on important regulatory and legal differences among EU nations in providing MAR treatments and regulate embryo research, including different points of view on key matters such as the handling of embryos, heterologous fecundation and surrogates, and preimplantation genetic diagnosis (PGD).

## 2. Materials and Methods

PubMed and Scopus databases (from 1970 to January 2014) were used, searching the following key words: medically assisted reproduction/procreation, in vitro fertilisation (IVF), and legislation and European Union. These key words were searched alone or associated with each other.

Several books, nonindexed sources, and institutional websites have also been used. Of the 531 sources found, only 55 were considered appropriate for the purpose of the paper.

## 3. Results

Below is a summary of the legislation in the field of MAR and HER for the 28 European Union countries. In those states where there is a* legal vacuum*, the authors have reported, where present, the guidelines, ethical indications, or general laws which do not fully control this important area of medicine.

### 3.1. Austria (AT)

Austria's law on MAR is very restrictive. The Act on Reproductive Medicine (Fortpflanzungsmedizingesetz, June 4, 1992) permits the access to MAR procedures only to married or stable heterosexual couples and forbids any form of egg cell donation. Sperm donation is forbidden for single women or lesbian couples, only those couples where the male is sterile may have access, but not in association with IVF [3].

This means that access to IVF is reserved for heterosexual married or stably living together couples, using their gametes. According to this Act, the mother of any progeny resulted after IVF is that woman who carried the pregnancy, rejecting, in this way, surrogate motherhood activities. The Act on Reproductive Medicine bans the use of human embryos for research purposes in any form [3, 4].

In 1998, two married Austrian couples affected by infertility complained about this regulation and they appealed to the Constitutional Court, sustaining that the ban of IVF with donated sperm or egg cells violated the right to form a family guaranteed by the European Convention on Human Rights. In 1999, the Court declared that even if the Act restricted the applicant's rights, it was justified taking into account the moral and ethical implications and the best interests of the child to be. Consequently, in 2000, the couples applied to the European Court of Human Rights, and the First Section in 2010 stated, in SH and Others v. Austria [5], that the Austrian regulation violated art. 14 and art. 8 of the Convention. Austria claimed that even though the right to respect for private life comprehends the right to reach the wish for a child; the State is not obliged to authorize any procedure aimed at procreation. Austria's legislation was formulated to avoid the creation of “unusual” personal relations (e.g., a child with two biological mothers). Austria claimed that the aim of the law has been to avoid “exploitation and humiliation of women, especially those from economically disadvantaged backgrounds,” who may be pressured to donate egg cells to other infertile women so as to receive IVF treatment which they could not afford otherwise.

The Grand Chamber of the European Court of Human Rights on November 3, 2011, reversed the judgment of the First Section and concluded that the restrictive Austrian assisted reproduction regulation is not contrary to the Convention [6].

Regarding HER, the Act on Reproductive Medicine (Fortpflanzungsmedizingesetz) 2004 firmly prohibits the use of human embryos for any other purpose than assisted reproduction. Therapeutic and reproductive cloning are therefore prohibited [7].

### 3.2. Belgium (BE)

MAR procedures in Belgium are regulated by two main laws, which are the law on medically assisted procreation and the destination of surplus embryos and gametes (loi relative à la procreation médicalement assistée et à la destination des embryons surnuméraires et des gametes) [8] of July 1, 2007, and the law regarding research on in vitro embryos (loi relative à la recherche sur les embryons in vitro) [9] of May 2, 2003.

Belgium has tolerant legislations in the field of MAR.

Section 10 states that unimplanted embryos can be cryopreserved (for 5 years according to art. 17, and art. 18 provides a possible extension of this period) taking into account possible future medically assisted insemination. If cryopreserved embryos are not implanted within limits, they can be destroyed, used for scientific purposes (law regarding research on in vitro embryos), or donated.

Parents, who have undergone MAR, have to sign an agreement with the clinic for the use of the unimplanted embryos in the case of separation, divorce, death of one of the parents, and so forth (art. 13).

Postmortem implantation of cryopreserved embryos is allowed (art. 15), between six months and two years after the death of the parent. Section 22 allows the embryo's donation but prohibits sale.

Some age limitations regarding the removal of gametes, embryo implantation, and insemination are present in art. 4; these practices are available for women up to the age of 45 years, whereas the implantation of embryo is allowed for women under 47 years.

Regarding PGD, there are no explicit legal provisions, it is however implicitly regulated by the law on embryo research (2003) that allows this practice in case of a high risk of genetic transmission disease. Sex-specific embryo selection is exclusively permitted in order to avoid sex-linked diseases [9, 10].

The law regarding research on in vitro embryos regulates this field in Belgium. This type of research according to art. 3 can be practiced only with therapeutic purposes and the prevention or treatment of illness. It can be carried out on embryos within the first 14 days of development and creation of embryos for research is permissible when the research aim cannot be achieved using supernumerary embryos and when the law's conditions are carried out. In Belgium human cloning is prohibited (art. 6) [9, 10].

### 3.3. Bulgaria (BG)

Currently, MAR procedures in Bulgaria are covered by the Bulgaria Health Act of August 2004 [11] and by Order 28 on Assisted Reproduction Activities 2007 (amended July 29, 2011) of Bulgarian Ministry of Health [12].

According to art. 129 of Bulgaria Heath Act, “assisted reproduction shall be applied, where the condition of the man or the woman prevents the natural performance of their reproductive functions” [11].

MAR includes all those activities related to “the application of medical methods for fertilization of an ovum located inside or outside the body of the woman” and the extraction of an ovum from one woman and the implantation into the body of the same woman or into the body of another woman [11].

According to art. 134, “ova, spermatozoa and fertilized ova which have not been used for creating progeny may be provided to research, educational and medical establishments” in Bulgaria and abroad for “medical, research and educational purposes upon receipt of the informed consent in writing of the donor or, in the case of fertilized ova, by the two donors.”

The export and import of ova, spermatozoa, and zygotes is regulated by Sections 36, 37, and 38 of the Organ, Tissue, and Cell Transplantation Act [13].

Art. 135 prohibits the selection of the gender of the progeny, unless gender related hereditary diseases have to be prevented and moreover “any intervention aimed at modifying the human genome may be undertaken only for preventive or therapeutic purposes and not for the purposes of introducing the modification into the genome of the progeny.” The Section bans the reproductive cloning of people, including that for the purposes of donating organs, tissues, and cells [11, 12]. HER is not directly regulated but falls within this act, whereby experimentation on surplus IVF embryos is permitted, under the informed consent of the donors.

Regarding Ordinance 28 on Assisted Reproduction Activities, the main elements which introduce MAR availability and regulations are as follows: IVF can be performed to women up to 51 years old, without restriction for single or female couple; there are no limits on egg, sperm, or embryo freezing; the number of embryos implantation is individually established; PDG and sex-selection are allowed only in order to avoid hereditary disease.

Sperm and egg donation are permitted, though egg donors should have at least one live birth and a maximum of 5 live births for sperm donors [12].

### 3.4. Croatia (HR)

As a result of the Europeanization process which involved Croatia in recent years, the previous conservative law on medical reproduction of 2009 was replaced in 2012 by a more liberal act (law on medically assisted reproduction) [14]. In particular, the new law allows cohabiting couples and single women to have access to MAR treatments; gametes and embryos can be donated and cryopreserved up to five years, with the opportunity to delay this time limit. Gender selection of embryos is only permitted in the eventuality of sex-linked diseases. Surrogacy and cloning are prohibited [14].

### 3.5. Cyprus (CY)

Presently, there are no rules covering MAR treatments in Cyprus. The National Bioethics Committee of Cyprus (CNBC) [15] started on February 2007 the discussion for the preparation of an opinion on MAR, trying to support the legislator in the drafting of the future law.

The key points of this discussion are below reported.

About the legal status of embryo the members of CNBC have expressed two opinions: according to the first one, “the embryo is created with the prospect of developing into a human being and it should be protected. The Cypriot legislation on the MAHP should have such provisions as to enforce the requirements of Section 18 of the Oviedo Convention and provide adequate protection to the embryo in vitro procreated for parental projects. Such adequate protection could be possible through transparent procedures for the applications of MAHP and prohibition of the procreation of supernumerary embryos in vitro in each parental project.” The second option is still under discussion.

According to the members of CNBC, MAR procedures should be used by “married couples with either ecclesiastical or political marriage.” The CNBC rejects the use of MAR procedures for homosexual couples and single parents.

About the possibilities of using surplus embryos in vitro, the CNBC believes that “only one embryo in vitro should be created in each parental project. Exception could be made for women aged above 40 years old, in this latter case, 2 or 3 embryos could be created and implanted in the uterus.”

Regarding the use of PDG methods, the CNBC did not adopt any unanimous positions.

HER using surplus IVF embryos is allowed if adequate protection of the embryos is ensured [16].

### 3.6. Czech Republic (CZ)

In Czech Republic, MAR is regulated by law number 227/2006 on research on human embryonic stem cells and related activities and on amendment to some linked acts [16, 17].

Section 3 of the Law states that research on human embryonic stem cells can be carried out only after authorization of the Ministry of Education, Youth, and Sport “on imported lines, provided that they were obtained from human embryos in such a way that does not object to the Czech legislation or legislation of the country of origin; their import is finalized only for research purposes which cannot lead to the creation of a new human being.”

Section 20 bans the following procedures: “interventions leading to creation of a human embryo for purposes other than implantation into a woman's body,” the import or export of a “human embryo or larger number of human embryonic stem cells or their lines inconsistent with a separate regulation,” the implantation of a generated human embryo into the uterus of another animal species, the implantation of “human genome into the cells of another animal species or vice versa, or the manipulation of human embryonic stem cells during their research in a way leading to the creation of a new human individual.”

In Czech Republic, “genetic examinations of embryos are permitted only in defined indications in order to exclude risks of serious genetically conditioned diseases and defects with embryos before they are implanted into the cavity of the uterus.”

Only married couples affected by infertility can have access to MAR treatments after that both of them have expressed their written consent.

Assisted reproduction can be applied in fertile age women and only homologous fecundation is allowed; Section 27 states that “donor of gametes for assisted reproduction purposes shall mean a person forming the infertile couple.” Only adult women from 18 to 35 years can donate their eggs for MAR purposes and men from 18 to 40 years can donate their sperm.

It is not allowed to choose the sex of the child during medical fertilization, except in the case of sex-linked genetic diseases [16, 17].

### 3.7. Denmark (DK)

In Denmark, MAR treatments are regulated by law number 460 of June 1997 on artificial fertilization in connection with medical treatment, diagnosis, and research [18], but in 2006 a new law (number 535 of June 8, 2006) [19] amended the previous law, leading to the following changes: the extension of treatment at local hospitals; the possibility for unmarried women and lesbians to have access to MAR; the evaluation of parental incompatibility and the prolongation of the storage period for cryopreserved eggs from 2 to 5 years. Order number 1724 of December 21, 2006, on artificial fertilization provided the authorization of inseminated oocytes for research purposes.

About preimplantation genetic diagnosis (PGD), law number. 240 of April 5, 2004, amended Section 7 of law 460/1997, stating that the National Board of Health can permit in definite cases the use of PGD in association with artificial fertilization. Such cases are related to the treatment of children with potential lethal diseases.

About storage and donation of sperm, it is possible to donate sperm to the donor's partner (homologous fecundation) or to another woman (heterologous fecundation) or for research purposes. In those cases of homologous fecundation, in the eventuality of death of the donor, his sperm cannot be used anymore and it must be destroyed.

For heterologous fecundation the sperm donor must remain anonymous and the donor cannot receive any information that can allow identification of the couple or the child (Chapter 2, law number 460/1997). HER is allowed on surplus embryos after consent of the donors.

About biomedical experimentation on fertilized human oocytes and gametes intended for use in fertilization, law number 460 of June 10, 1997 (Sec. 25), states that “research may only be carried out for the purpose of improving IVF or similar techniques; in order to improve techniques for the genetic testing of a fertilized oocyte with a view to establishing the possible presence of a serious hereditary disease or an important chromosome abnormality (preimplantation diagnosis); if the purpose of experiments involving the use of fertilized oocytes and stem cells derived there from is to obtain new knowledge that could improve the possibilities of treating diseases in human beings; the removal and fertilization of an oocyte in order to carry out experiments other than those referred to in subsection 1 shall be prohibited” [18, 19].

### 3.8. Estonia (EE)

The law “Assisted fertilization and Protection of the Embryo” (RT I 1997, 51, 824) [20] was promulgated in Estonia in July 1997 and it is composed of 5 sections and 36 paragraphs and it regulates the following procedures: artificial insemination, IVF, and embryo research.

According to this law, for MAR procedures is necessary a written agreement of both members of the couple who undergo these types of treatments. The development of embryos is permitted up to 14 days, whereas they can be cryopreserved up to 7 years.

In Estonia, according to this law, the donation of sperm, eggs, and embryos is permitted and anonymous. Taking into account the Estonian population (less than 1.3 million), from a single sperm donor is not possible to have more than 6 children. It is not allowed to transfer more than 3 embryos [20].

The following procedures are banned: the use of fresh donor sperm (only cryopreserved after control of the donor for sexually transmitted diseases after 6 months); postmortem fecundation using the stored sperm of the husband after his death; cloning of human beings. Regarding research on embryos, it is allowed only with the authorization of Committee of Bioethics [20].

### 3.9. Finland (FI)

Finland's law on MAR procedures is regulated by the Act on Assisted Fertility Treatments (1237/2006) [21]. “This Act applies to the provision of assisted fertility treatment in which a human gamete or an embryo is placed in a woman for the purpose of creating a pregnancy. This Act also applies to the donation and storage of gametes and embryos for use in assisted fertility treatment.”

Section 4 of the act provides some restrictions about the use of gametes and embryos: genetically manipulated gametes and embryos, cloned embryos and gametes, and embryos, which have been used in research, cannot be implanted.

In Section 5 it is stated that “the health of the child to be born may be influenced by selecting gametes or embryos that have been verified to be free of serious disease. Determination of the child's sex maybe influenced if the gametes used in the assisted fertility treatment are the couple's own and the child born from these gametes would be at substantial risk of serious disease if the child were to be of the other sex.” Stored gametes and embryos have to be destroyed in case of death of the person whose gametes are concerned or in case of death of the donor (Section 4). Instead of destroying gametes and embryos, they can be used “for another legal purpose if a consent for such use has been obtained from the person or persons whose gametes are concerned or whose gametes gave rise to the embryos” [21].

Section 8 of the act bans MAR treatments if “the written consent of the person receiving the treatment has not been obtained; either party of the couple receiving treatment is married to a third person; pregnancy would pose a substantial risk to the health of the woman or the child due to the age or health of the woman; a person consenting to the provision of assisted fertility treatment has withdrawn consent or died” [21].

Gametes can be donated by persons over the age of 18 who pass a physical examination and who do not have “any serious inherited disease or any communicable disease that may cause a serious illness to the woman receiving assisted fertility treatment or to the child who may be born as a result of assisted fertility treatment” (Section 13).

Section 20 states that “a couple may donate extra embryos created for use in their own assisted fertility treatment for use in the assisted fertility treatment of another. The consent of both the woman and the man is required for donation, and both shall be considered donors.”

The act bans the production of embryos for research purpose (Section 13) and prohibits “research on embryos and gametes for the purpose of developing procedures for modifying hereditary properties… unless the research is for the purpose of curing or preventing a serious hereditary disease” (Section 15) [21].

The laboratories that perform embryo research need a license from the National Authority for Medicolegal Affairs and a written consent from both gamete donors is required [21].

### 3.10. France (FR)

In France, MAR is regulated by Law on Bioethics of 2011 (LOI n° 2011-814 du 7 juillet 2011 relative à la bioéthique) [22], Bioethics Law 2004-800 (August 6, 2004) [23], and Law 1994-654 [24] on the Donation and Use of Elements and Products of the Human Body, Medically Assisted Procreation, and Prenatal Diagnosis of July 1994.

The Bioethics Law of 2004 and the 2011 amendment prohibit the creation of embryos for research purposes, but research on supernumerary IVF embryos up to 8 days is permitted after written consent of parents who do not require them for further inseminations. Embryos unsuitable for reimplantation or storage can be used for research purposes. As required by the Bioethics Law of 2004 (Chapter II, Section 16) [23] all research projects have to be authorized by the French Biomedicine Agency which requires four conditions: “the research is scientifically relevant; the research is likely to allow major medical advances; it is expressly established that the research cannot be performed unless cells derived from embryos are used; the research project respects French ethical principles for research on embryos and embryonic stem cell lines.” Embryonic stem cells can be imported into France, subject to prior approval by the Biomedicine Agency.

MAR procedures are available only for heterosexual married couples or heterosexual couple who have been living together for at least two years and both partners must be alive and in procreation age (in vitro fertilization is financed by French Health System for women within 43 years). Those couples, who can transmit disease to offspring with natural procreation, can have access to MAR and, in this latter case, the couple can receive an egg or sperm from an external donor [22, 23].

### 3.11. Germany (DE)

In Germany, MAR is regulated by the Law on the Protection of Embryos (Embryonenschutzgesetz) [25] of 1990 and the act ensuring protection of embryos in connection with the importation and use of human embryonic stem cells of 2008 [26]. There are also some Guidelines of the German Medical Association about the Performance of Assisted Reproduction.

The German act bans to transfer more than 3 embryos per cycle to a woman or to fertilize more than three ova per cycle by gamete intrafallopian transfer. All forms of trade in embryos are prohibited. Section 3 of the act prohibits the selection of gender whereas Section 5 prohibits human cloning and the creation of chimeras and hybrids.

The Guidelines of the German Medical Association on the Performance of Assisted Reproduction govern when it is possible to use homologous or heterologous in vitro fertilization.

According to these guidelines, cryopreservation of ova in the pronucleus phase is allowed for the treatment of infertile women, whereas cryopreservation of embryos is only permitted if the transfer of the embryo was not possible during the treatment cycle. Cryopreservation of ova is allowed.

The German act and the guidelines try to avoid the creation of surplus embryos, but for those cases in which it happened there are no specific rules on how to manage them.

German law in the field of MAR does not provide age limits for using this type of treatments; only the guidelines recommend taking into account the age of the woman for the number of embryos which are going to be implanted to avoid multiple pregnancies.

German law allows PGD before embryo's intrauterine transfer, in order to prevent genetically transmitted diseases. The use of embryos for research is heavily restricted in Germany under the Embryo Protection Act of 1990 (Section 2), which makes the derivation of embryonic stem cell lines a criminal offence.

### 3.12. Greece (GR)

In Greece, the first law in the field of MAR was enacted in 2002 (law 3089/2002) [27] and then another law (law number 3305/2005) [28] was voted in 2005, which regulates MAR treatments. According to the law of 2005, MAR represents a “medical necessity” in case of limitation in natural procreation or if there is a risk to transmit a sexually transmitted disease or a hereditary disease to the child.

Women up to 50 years old can have access to MAR after providing her written consent. In case of cohabiting couples or single women, a notarial consensus is necessary. It means that unmarried couples and single women can have access to assisted reproduction.

In Greece, the following procedures are banned: cloning of a human being and the choice of sex (only in case of sex-linked diseases or transmitted sexual diseases, it is allowed).

Heterologous fecundation is permitted and when for fertilization the sperm of an external donor is used; because the husband has given his consent for the treatment, he has the fatherhood of the child, even if the child does not draw its origin from him.

Surrogate motherhood in Greece is allowed when the woman is not able to carry the pregnancy, either if she has her own ovum or she needs the uterus of another woman or if she needs both uterus and ovum of another woman. A written agreement must be done between the couple or single woman who wants the child and the surrogate mother. It is necessary that both women have to be resident in Greece.

Postmortem fertilization is allowed if the man gave his written consent for the use of sperm after death. In this case artificial fecundation can be performed between six months and two years after the donor's death [28].

Section 1459 of law 3089/2002 [27] on medically assisted human reproduction states that fertilized ova can be used for research after written consent of the parts or can be donated to another couple or destroyed. If there is no declaration about their fate, following 5 years of cryopreservation, they can be destroyed or used for research purposes. Research on embryos that are over fourteen days old is prohibited. Human reproduction with the methods of cloning is expressly prohibited in Section 1455 [27].

### 3.13. Hungary (HU)

In Hungary, MAR procedures are covered by the Act on Health, Chapter IX, 20/2007 [29], and by Ministerial Decree 30/1998 [30].

Married couples and heterosexual stable couples can have access to MAR. Single women can be treated in case of proven infertility. About homosexual couples, lesbians can have access to MAR and they are considered single women, whereas male homosexual couples cannot receive any treatments because IVF surrogacy is not allowed in Hungary.

All women within the age of 45 years can have access to MAR; in case of single women, they can receive heterologous fecundation with an anonymous sperm donor.

In Hungary according to the present law, the donation of sperm, eggs, and embryos is permitted and anonymous. Sperm donors must undergo a rigorous screening process and its sperm can be used up to a maximum of 4 live births; egg donors must be under 35 and must have given birth to at least one healthy child. Egg donation from a relative is acceptable.

About embryo transfer policies, it is not allowed to transfer more than 4 embryos.

Embryos can be cryopreserved up to 10 years, but additional storage is allowed after certification of their conservation status and a written statement. Sex selection is permitted in order to avoid an inheritable disease, while genetic pathways can only be selected for preventing or treating possible genetic illnesses. Hungarian MAR legislations do not permit the use of sperm after donor's death. HER is allowed for surplus IVF embryos up to 14 days, whereas creation of in vitro embryos for research purpose and reproductive and therapeutic cloning is strictly prohibited.

### 3.14. Ireland (IE)

Currently, there are no rules covering MAR treatments in the Republic of Ireland.

In the absence of regulatory legislation focusing on MAR, the “Guide to Professional Conduct and Ethics for Registered Medical Practitioners” represents the only guidance for medical practitioners in Ireland. It has been published first in 2004 and more recently in 2009, which represents the last version [50, 52].

In the 2009 version “assisted human reproduction treatments, such as IVF, should only be used after thorough investigation has shown that no other treatment is likely to be effective.”

Regarding “gamete donation”, Medical Council guidelines state that “if you offer donor programs to patients, you must consider the biological difficulties involved and pay particular attention to the source of the donated material. Such donations should be altruistic and non-commercial. You should keep accurate records for future reference.”

Furthermore, according to 2009 Guidelines, MAR services “should only be provided by suitably qualified professionals, in appropriate facilities, and according to international best practice” [50, 52].

### 3.15. Italy (IT)

In February 2004, the Italian Parliament approved the law 40/2004 (rules in the field of medically assisted reproduction), which regulates MAR treatments [31, 32, 53, 54].

In art. 4 it is stated that the access to assisted reproduction techniques is limited to those cases of infertility or unexplained infertility documented with medical procedure as well as cases of sterility or infertility ascertained and certified by a medical act.

It is forbidden heterologous fertilization (art. 4) and only couples of the opposite sex, married or cohabiting, childbearing age and both living, can have access to MAR treatments.

Regarding research on human embryos (art. 13), experimentation on each human embryo is banned. Clinical and experimental research is allowed after providing that only therapeutic and diagnostic aims are pursued for the protection of the health and development of the embryo. The following procedures are not allowed: any form of eugenic selection of embryos and gametes; cloning by nuclear transfer or embryo early splitting or ectogenesis both for procreation or research purposes; and the fertilization of a human gamete with a gamete of different species and the production of hybrids or chimeras.

About PGD, a restrictive interpretation of Section 13 has led to denying infertile couples with genetic diseases the right to seek PGD; however the Constitutional Court has many times expressed the unconstitutionality of the rule [53, 54].

On May 2009, the Italian Constitutional Court (N°151/09) [32] banned some restrictions set out in the 40/2004 law, declaring the constitutional illegitimacy of subparagraphs 2 and 3 of Section 14 that banned the cryopreservation and suppression of embryos and the fertilization of more than three oocytes at the same time during an IVF treatment and it obliged the implantation of all embryos obtained. Now, Italian reproductive specialists are able to decide how many embryos will be best in order to achieve a pregnancy (removing the upper limit of three) and the duty to implant all embryos together has been removed, trying however to limit the number of cryopreserved embryos, which cannot be destroyed or donated. For this reason, even though the Italian Constitutional Court removed some restrictions imposed by law 40/2004, problems such as the storage of cryopreserved embryos for an indefinite time remain unsolved [32, 53, 54].

More recently (April 9, 2014) [33], the Constitutional Court has legitimized the heterologous artificial insemination, declaring unconstitutional the sections of law number 40 of February 19, 2004, which prohibited the heterologous fertilization: (a) Section 4, paragraph 3: “It is forbidden the use [*sic*] of techniques of medically assisted procreation of heterologous type”; (b) Section 9, paragraphs 1 and 3, which included the prohibition of the disclaimer of paternity and the anonymity of the mother; (c) Section 12, paragraph 1, which included penalties for anyone who uses for procreation purposes, gametes of subjects outside the applicant couple.

### 3.16. Latvia (LV)

Latvia's law on MAR procedures is regulated by the Sexual and Reproductive Health Act of 2002 [34]. “The purpose of this law is to define legal relations within the field of sexual and reproductive health with the aim to protect unborn life and the sexual and reproductive health of every person.” Section 13 of Chapter 5 defines “medical impregnation” as an artificial fusion of male and female gametes. Only heterosexual couples or women on the basis of a written application submitted to the medical treatment institution may have access to MAR treatments. In vitro fertilization is performed “using the gametes of a donor or of the genetic parents.” Section 15 bans the fusion of human and animal gamete nuclei with the aim to obtain fertilization; to introduce a human embryo into the system of a primate or animal of any other species; to obtain a human embryo for scientific research and its use as a tissue and organ donor; to use gametes of the donor or the embryo for commercial purposes; to choose the sex of the child during medical fertilization, except in the case sex-linked genetic diseases; and to implant at the same time more than three fertilised ovaries in a woman's body. Human cloning is prohibited.

About the donation of gametes, the donor must be a healthy person: male in the age of 18 to 45 years and female in the age of 18 to 35 years (Section 17).

Gametes stored for more than 10 years can be destroyed as well as defected embryos. In the event of the death of the donor, only if the donor has given written consent to the use of the gametes after his/her death, they can be used; otherwise gametes must be destroyed. Latvia has no specific regulations about HER but allows some research on supernumerary IVF embryos [34].

### 3.17. Lithuania (LT)

Lithuanian law in the field of MAR is very severe. The most important legislation is represented by the Law on Ethics of Biomedical Research [35], first promulgated in 2000 and amended in 2007, which states that “human embryos may be subjects only of clinical observations (non-invasive investigations). Other clinical investigations involving human embryos and their creation for purposes of biomedical research shall be prohibited. Human embryos may be subjected to such biomedical research where the medical risks for the embryo are not disproportionate to the potential benefits.” Equally, the law states that the cloning of a human being is banned and also the import and export of tissues of a human embryo, stem cell of human embryo, and lines are prohibited [35]. The Law on Donation and Transplantation of Human Tissues, Cells, and Organs number I-1626 of November 19, 1996 [36], controls the storage and use of tissue (adult) stem cells for transplantation purposes.

### 3.18. Luxembourg (LU)

Presently, there is no law regulating MAR procedures in Luxemburg and these practices are based on human sense, on ethical commission guidelines, and only partly on French bioethical law July 1994 [23]. There is no legislation on stem cell research.

### 3.19. Malta (MT)

There is no Legislation in place.

### 3.20. Netherlands (NL)

In the Netherlands, the Embryos Law of 2002 [37] regulates MAR procedures and bans both human reproductive cloning and the creation of hybrids and chimeras. The law makes a distinction between cloning for reproductive purposes and research-oriented somatic cell nuclear transfer (SCNT), instituting a five-year moratorium on SCNT. The creation of human embryos for research purposes is illegal under the law. A 2007 reevaluation of the policy by the Dutch cabinet ended with the existing policy being left in place for the foreseeable future [37].

### 3.21. Poland (PL)

In Poland there is no specific legislation in the field of MAR.

Some provisions regarding MAR treatments can be found in the Medical Profession Act of December 5, 1996, the Health Care Institutions Act of April 15, 2011, and the Medical Ethics Code [51, 55].

According to art. 26 of the Act on the Medical Profession, unborn children cannot be involved in scientific experiments. No mention is made regarding therapeutic experiments, which makes the practice permissible, even if there is no regulation on this subject. Poland has no legal regulations relating to the prohibition of human cloning but, in 1999, Poland signed the Additional Protocol to the Convention on Human Rights and Biomedicine on the Prohibition of Cloning Human Beings of the Council of Europe and at the same time the Code of Ethics for Medical Doctors bans their participation in any procedure aimed at human cloning (art. 39a). There is no legislation on stem cell research [51, 55].

### 3.22. Portugal (PT)

In Portugal, law number 32/2006 [38] regulates MAR treatments and research on embryo.

MAR techniques have to be considered to be subsidiary and not an alternative method of procreation. These procedures may be used only in the case of infertility or in order to treat a serious disease or prevent the risk of transmission of genetic, infectious, or other diseases (Sec. 4). MAR may only be practiced in public or private centers expressly authorized for the purpose by the Ministry of Health (Sec. 5). Only persons who are married or who have been partners in an equivalent relationship for at least two years, who are not of the same sex, who are at least 18 years old, who are not subject to guardianship, and who are not mentally disabled may have recourse to MAR (Sec. 6). The following are prohibited: reproductive cloning; the use of MAR techniques for sex selection, except in the case of sex-linked genetic diseases; the use of MAR techniques to create chimeras or hybrids; and the use of preimplantation genetic diagnosis techniques in the case of multifactorial diseases where the predictive value of the genetic test is very low (Sec. 7). Surrogate motherhood whether for payment or otherwise is not recognized; the woman who undergoes pregnancy being deemed, for all legal purposes, to be the mother (Sec. 8). The use of MAR techniques for the express purpose of creating embryos for research is prohibited. Scientific research with embryos is, however, permitted for the purposes of prevention, diagnosis, or therapy, the improvement of MAR techniques, and the constitution of stem cell banks for transplantation or other therapeutic objectives. Such research is subject to the decision of the National Council for Medically Assisted Procreation (Sec. 9). Use may be made of donated sperm, oocytes, and embryos where, in the light of current scientific knowledge, it is not possible for a woman to become pregnant by any other means and where the quality of gametes can be assured (Sec. 10) [38].

### 3.23. Romania (RO)

In Romania, in order to receive MAR treatments, the man and the woman need to fulfill the following conditions, according to the law 63/2012 [39], art. 21: “to be alive, the artificial insemination of the woman with the sperm of the deceased husband is prohibited; to be at the biological reproductive age; to be married or able to probe a common life together of at least 2 years; to consent previously to the transfer of embryos or to artificial insemination.”

According to art. 13 the access to MAR services is “granted to any woman or man suffering from sterility, that cannot be treated with a classic method of treatment or surgical intervention,” providing practical examples.

In Section 14 are banned some activities in the area of MAR, among these are “abusive production of embryos; genetic manipulation on embryos; post-mortem insemination; illegal donation of embryos; gametes trafficking; collection of gametes without consent; mixing gametes; selective abortion of embryos of a certain sex.

The allowed techniques of MAR according to Romanian law (art. 15) are (a) artificial insemination; (b) in vitro fecundation; (c) transfer of embryos.

About the procedure of artificial insemination (art. 16) it can be performed by “the insemination with the sexual cell of the partner or insemination by using the cell of a donor, which applies in the case of genetic or communicable diseases of the partner.”

Human cloning is prohibited in Romanian law [39].

### 3.24. Slovakia (SK)

Slovakia's law on MAR procedures and human cloning is very severe. According to Law 277/1994 [40] on Health Care, research “without therapeutic purpose” on human embryos and foetuses is prohibited. Taking into account that there are no other requirements concerning human embryos in the present legislation, research “with therapeutic purpose” should be allowed. The same law bans any form of human cloning, with and without therapeutic purposes. This prohibition is also supported by the Slovakian Penal Code, Law 140/1961, which affirms that “any intervention aiming to create a human being in any stage of its formation, which is genetically identical to another human being whether living or dead, is prohibited.” The violation of these laws results in financial penalties and custody up to 8 years. There is no specific legislation on stem cell research; however, Slovakia ratified the Convention on Human Rights and Biomedicine and of the Additional Protocol on the Prohibition of Cloning of the Human Beings and banned all human cloning, as stated by law number 277/1994 on health care [40].

### 3.25. Slovenia (SI)

In Slovenia, MAR is regulated by the Law on Biomedically Assisted Fertilisation number 70/2000 [41]. According to this law (art. 4), “early embryo” is considered to be embryo which progresses outside the uterus during the first 14 days. Art. 38 allows the use of early embryos and cryopreserved embryos which may no longer be used for reproduction, for research purposes, but the specific creation of human embryos for research is prohibited according to art. 33 of law 70/2000 and the art.18 of the Oviedo Convention. Art. 33 bans embryo's in vitro development over 14 days. Art. 33 clearly forbids the creation of embryos genetically identical to another human being, living or dead. This means that also cloning for therapeutic purpose is illegal [41].

### 3.26. Spain (ES)

In Spain, MAR is regulated by the Law on Assisted Human Reproduction Techniques, number 14/2006 (May 27th, 2006) [42], and the Biomedicine Law 14/2007 (July 3, 2007) [43].

Chapter two of the 2006 Act sets out who can have access to MAR and the modalities of access; art. 5 states that the donation of gametes and preembryos for the purposes authorized by this act is nonremunerative, formal, confidential and with mutual agreement between the donor and the authorized centre. The donation must never be regarded a lucrative or commercial process and must never be promoted by compensation offers or economic benefit. The Minister of Health, with authorization from the Comisión Nacional de Reproducción Humana Asistida (National Committee of Assisted Human Reproduction), will periodically set essential conditions to ensure that the respect for donation being a nonlucrative action is maintained. Donation has to be anonymous, and gamete banks must guarantee the confident donor's identity. Individuals born as a result of assisted reproduction have the right to obtain general information about donors, but not their identities.

No more than 6 children can be conceived with the sperm of the same donor. All women (both single and married, regardless of their sexual orientation) older than 18 years can receive MAR treatment after written consent. If the woman is married, it is necessary also to have the written consent of her husband (art. 6).

Section 8 sets out the* legal determination of filiation *and it states that the mother and father after having given their written consent cannot reject the parental rights of the unborn.

Regarding postmortem fecundation (art. 9) it can be performed up to 12 months after the husband/partner death, only if he has given written consent to use its sperm after death.

Surrogacy is not recognized in Spain; art. 10 states that a “contract drawn up in reference to a paid or unpaid surrogate pregnancy regarding a woman who renounces maternal filiation in favour of the contracting party, or a third party, will be null and void” and filiation of children born through surrogacy is determined by birth.

According to law 14/2006, gametes can be used for research purposes or experimentation but they cannot subsequently be implanted into a woman or be used to create preembryos for reproductive purposes (art. 14).

Section 15 states that the research or experimentation using embryos originally created for reproduction purposes, but subsequently no longer required for such purposes, is considered ethically acceptable if written consent of the couple is given, or woman alone when applicable, after they have undergone a detailed explanation of the aims of the investigation and its implications. The preembryo must not have been developing in vitro for more than 14 days after fertilization.

Research or experimentation must be carried out following a correctly presented project plan. The plan must be approved by the competent health authorities, and if the investigation is related to embryonic development, it must be authorized by the National Committee of Assisted Human Reproduction. The law bans reproductive cloning [42, 43].

### 3.27. Sweden (SE)

In Sweden, the Genetic Integrity Act number 351 of May 18, 2006 [44], regulates MAR treatments. Other Rules are represented by the Health and Medical Services Act number 763/1982 [45], which provides provisions on patients' self-determination and on respect for the equal dignity of all human beings within health and medical services. The Biobanks in Medical Care Act number 2977/2002 [46] sets outs that biological material has be collected, stored, and employed, taking into account the integrity of the person.

The purpose of the Genetic Integrity Act number 351/2006 [44] is to establish “provisions on restrictions on the use of certain biotechnology developed for medical purposes and on certain legal effects of such use.”

Regarding PGD, in Section 2 of Chapter 4 it is stated: “Pre-implantation genetic diagnosis may only be used if the man or woman has a predisposition towards a serious monogenetic or chromosomal hereditary disease, which entails a high risk of having a child with a genetic disease or impairment. The treatment may not be used to choose characteristics, but only be aimed at preventing the child from inheriting the predisposition towards the disease or impairment in question.” To access to PGD, the authorization of the National Board of Health and Welfare is necessary [45, 46].

In Sweden there is a remarkably strict regulation about the “consent” for the use of human eggs for research purposes. In case of “fertilisation outside the body” it is required that “the woman or man who is not the donor of egg or sperm in the couple being treated has been informed of the purpose of the measure and thereafter given her or his consent.”

Research on embryo can be carried out up to 14 days after fertilisation and then it has to be destroyed. A fertilised egg or an egg used for somatic cell nuclear transfer can be cryopreserved up to five years, but the National Board of Health and Welfare can prolong this period. Embryos, sperm, and eggs used for research purposes can no longer be used for implantation or fertilization.

Chapter 6 of Act number 351/2006 establishes that “insemination may be carried out only if the woman is married or cohabiting. Written consent for insemination is required from the spouse or cohabitee” [44].

Eggs or sperm from a deceased donor cannot be used for fertilisation. About the age to access to MAR in Chapter 7 among conditions for treatment, it is only stated that “a donor of an egg or sperm shall be of age” [45].

### 3.28. United Kingdom (UK)

In UK, MAR treatments and human embryo research are regulated by the Human Fertilization and Embryology Act of November 13, 2008 [47], which amended the Human Fertilization and Embryology Act 1990 [48] and the Surrogacy Arrangements Act 1985 [49].

The Human Fertilization and Embryology Authority (HFEA) is responsible for applying the regulations of the Human Fertilization and Embryology Act and for licensing both IVF clinics and scientists carrying out research on human embryos. As stated in Sections 3 and 4 of the act, it is considered illegal to store or use embryos or gametes without a license provided by the HFEA.

In Section 3 (prohibitions in connection with embryos) it is stated that only “permitted” embryos can be implanted in the uterus of a woman. An embryo is “permitted” if it has been created by the fertilization of an egg “produced by or extracted from the ovaries of a woman, and whose nuclear or mitochondrial DNA has not been altered” and sperm “produced by or extracted from the testes of a man, and whose nuclear or mitochondrial DNA has not been altered.” Exceptions can be accepted only “to prevent the transmission of serious mitochondrial disease” [47].

For research purposes it is necessary to submit a research project to the HFEA, which will verify if the project is suitable for the purposes specified by the HFE Act. These purposes include increasing knowledge about serious medical conditions, developing treatments for serious medical conditions, advancing the treatment of infertility, increasing knowledge about the causes of miscarriage, developing more effective contraception techniques, developing methods for detecting genetic or mitochondrial abnormalities in preimplantation embryos, and increasing knowledge of embryonic development. For research purposes the following can be used: unemployed IVF embryos, embryos created by IVF explicitly for research, embryos created by somatic cell nuclear transfer, “admixed embryos” including hybrids (created from human and animal gametes), “cytoplasmic hybrids” (created by somatic cell nuclear transfer, using human nuclei and animal oocytes), transgenic human embryos (created by introducing animal DNA into a human cell), chimeric human embryos (created by introducing one or more animal cells into a human embryo), or any other embryos that contain both human and animal DNA, but in which animal DNA is not predominant. Research on embryos is limited within the first 14 days [47].

About parenthood in cases of MAR, in Section 27 it is stated that the mother is “the woman who is carrying or has carried a child as a result of the placing in her of an embryo or of sperm and eggs, and no other woman, is to be treated as the mother of the child.” For married women at the time of treatment, in case of fecundation with the sperm of an external donor (heterologous fecundation), the husband has to be considered the father of the child unless it is shown that he did not consent to the treatment. Regarding postmortem fecundation, a man whose sperm has been used after his death must be considered the father of the child only if he has given written consent to use his sperm after death.

Sections 42 and 43 of the act state that a woman in a civil partnership with another woman can receive IVF treatment or a woman who has consented to be a parent of the child born as a result of IVF to be the “other parent” of the child [47].

About “surrogacy,” the Human Fertilization and Embryology Act 2008 [47] amend the Surrogacy Arrangements Act 1985 [49]. According to the present law to advertise for a surrogate mother or for third parties to negotiate a surrogacy arrangement on a commercial basis is illegal. The Human Fertilization and Embryology Act 1990 made for the first time a legal process which allowed married parents to reassign legal parenthood to them and obtain a parental order. The 2008 Act extended the right to apply for a parental order to unmarried and homosexual couples (Sections 33–54, issued from April 6, 2010, forwards).

## 4. Discussion and Conclusions

European legislation in the field of medically assisted reproduction and human embryo research is rather different in each country of the Union (Table 1), and not all European countries have specific legislation (Table 2). These laws derive from different origins ranging from an extremely prohibitive legislation (e.g., in IT, DE, LT, and AT), versus a cautious regulatory approach in DK, SE, and FR and a liberal regulatory system in the UK, ES, GR, and NL.

The most important differences regarding the handling of embryos and research, heterologous fecundation, surrogates, and PGD are summarized in Table 3.

About the management of embryos, in some European countries they can be cryopreserved for a defined period: for example, 2 years (with possible extension to 5) in DK, 5 years in BE (with possible extension) and GR, 7 years in EE, 10 years in HU, LV, and SE; in Germany, the German act and the guidelines try to avoid the creation of surplus embryos, but for those cases in which it happens there are not specific rules on how the manage them. In Italy, the Constitutional Court (N°151/09) banned some restrictions set out in the 40/2004 law and, now, Italian reproductive specialists are able to decide how many embryos will be best in order to achieve a pregnancy (removing the upper limit of three) and the duty to implant all embryos together has been removed, trying however to limit the number of cryopreserved embryos, which cannot be destroyed or donated (Table 3).

Regarding research on human embryos, in those EU countries where it is allowed, they make a distinction between the production of embryos for research purposes and research on existing embryos. Except UK (where unemployed IVF embryos, embryos created by IVF explicitly for research, and hybrid embryos created from human and animal gametes can be used) and those countries without a specific legislation in this field, the other EU members, which permit research on existing embryos no longer suitable for implantation (BE, CZ, EE, FR, GR, HU, LV, LT, NE, PT, SK, SI, ES, and SE), ban the creation of embryos specifically for research purposes. Among these countries, research on embryos for therapeutic purposes can be carried out up to 14 days of development. In AT and IT every form of experimentation on human embryos is banned (Table 3).

Presently, the scenario arising by comparing MAR and HER laws in the European Union is far from being uniform for the substantial differences highlighted among EU members; therefore a first issue that should be addressed is the role of EU and the other central bodies in regulating this field and how national and European laws could synergistically interact in order to pursue this aim.

Currently, those European citizens, belonging to Nations with restrictive legislations in this field, have a limited access to assisted reproduction techniques now available and according to the authors this phenomenon may generate a “treatment gap” in comparison to citizens of EU countries with a liberal regulatory system. Therefore a greater interaction among European member states is desirable in order to “harmonize” the positions adopted by each EU country, taking into account the growing expectations in people affected by infertility and the significant impact that MAR covers in all levels of society: cultural, religious, medical, and legislative.

Finally, it is interesting to underline how, in the judgment of the European Court of Human Rights in the case of SH and Others v. Austria [5], the Court considers this area “in which the law appears to be continuously evolving and which is subject to a particularly dynamic development in science and law,” to keep under review by the contracting states.

The recent decision of the Italian Constitutional Court on April 9, 2014 [33], which for the first time has legitimized the heterologous artificial insemination in Italy, declaring unconstitutional the sections of law number 40 of February 19, 2004, which prohibited the heterologous fertilization, represents therefore an emblematic example of this dynamic process, which is in constant evolution.

The Italian Constitutional Court has taken into account the arguments already mentioned the case of SH and Others v. Austria [5], and with a reference to Sections 8 (right to respect for private and family life) and 14 (prohibition of discrimination) of the European Convention on Human Rights [56] has taken a valid legislative support of its decision.

Medical science is in fact a constantly evolving matter; in particular, the development of knowledge involves the identification of new methodologies, which are able to offer the achievement of objectives previously unthinkable and therefore the law has to conform to this evolutionary process.

## Figures and Tables

**Table 1:** EU countries with laws in the field of MAR.

EU countries	Laws
Austria	The Act on Reproductive Medicine (Fortpflanzungsmedizingesetz) of June 4, **1992** [3]. Reproductive Medicine Act Amendment 2004 (Fortpflanzungsmedizingesetz geändert wird (Fortpflanzungsmedizingesetz-Novelle 2004) [7]

Belgium	Law on medically assisted procreation and the destination of surplus embryos and gametes (loi relative à la procreation médicalement assistée et à la destination des embryons surnuméraires et des gametes) of July 1st, **2007** [8]. Law regarding research on in vitro embryos (loi relative à la recherché sur les embryons in vitro) of May 2, **2003** [9].

Bulgaria	The Bulgarian Health Act (**2004**) [11]. Order number 28 of June 20, **2007,** on assisted reproduction [12]. Law on Transplantation of Organs, Tissues, and Cells (**2003**) [13].

Croatia	Law on medically assisted reproduction, **2012** [14]

Czech Republic	Law number **227/2006** on research on human embryonic stem cells and related activities [16, 17].

Denmark	Law number 460 of June **1997** on artificial fertilization in connection with medical treatment, diagnosis, and research [18]. Law number 535 of June 8, **2006** [18]. Order number 1724 of December 21, **2006** [18]. Law number 240 of April 5, **2004** [18].

Estonia	Law “Assisted fertilization and Protection of the Embryo” **1997** [20].

Finland	Act on Assisted Fertility Treatments (1237/**2006**) [21].

France	Bioethics Law number 814/**2011** [22]. Bioethics Law number 800/**2004** [23]. Law 654/**1994** on the Donation and Use of Elements and Products of the Human Body, Medically Assisted Procreation, and Prenatal Diagnosis [24].

Germany	Law on the Protection of Embryos (Embryonenschutzgesetz) of** 1990** [25]. Stem Cell Act (**2008**) [26].

Greece	Law 3089/**2002** [27]. Law number 3305/**2005** [28].

Hungary	Act on Health, Chapter IX, 20/**2007** [29]. Ministerial Decree 30/**1998** [30].

Italy	Law 40/**2004** [31]. Italian Constitutional Court (number 151/09) [32]. Italian Constitutional Court (April 9, 2014) [33].

Latvia	Sexual and Reproductive Health Act of **2002 **[34].

Lithuania	Law on Ethics of Biomedical Research, first promulgated in 2000 and amended in **2007** [35]. Law on Donation and Transplantation of Human Tissues, Cells, and Organs number I-1626 of November 19, **1996** [36].

Netherlands	Embryos Law of **2002** [37].

Portugal	Law number 32/**2006** [38].

Romania	Law 63/**2012** [39].

Slovakia	Law 277/**1994** on Health Care [40]. Slovakian Penal Code, Law 140/**1961** [40].

Slovenia	Law on Biomedically Assisted Fertilisation number 70/**2000** [41].

Spain	Law on Assisted Human Reproduction Techniques, number 14/**2006** [42]. The Biomedicine Law 14/**2007** [43].

Sweden	Genetic Integrity Act number 351 of May 18, **2006** [44]. Health and Medical Services Act number 763/**1982** [45]. Biobanks in Medical Care Act number 2977/**2002** [46].

UK	Human Fertilization and Embryology Act 2008 [47]. Human Fertilization and Embryology Act 1990 [48]. Surrogacy Arrangements Act 1985 [49].

**Table 2:** EU countries without specific laws in the field of MAR but only “guidelines,” ethical indications, or general laws.

EU countries	Guidelines or ethical indications
Cyprus	The National Bioethics Committee Of Cyprus (CNBC) **2007** [15].
Ireland	The Guide to Professional Conduct and Ethics for Registered Medical Practitioners (**2009**) [50].
Luxembourg	No legislation in this field.
Malta	No legislation in this field.
Poland	Poland's Medical Profession Act of **1996** [51].

**Table 3:** Comparison of EU laws in the field of MAR and HER.

Country	Eligibility criteria to access MAR	Type of fecundation			PGD	Gender selection	Destination of surplus embryos and gametes			
		Homologous	Heterologous	Surrogacy			Donation	Cryopreservation	Postmortem fecundation	Research on surplus embryos
Austria	Married or stable couples	Yes	No	No	Undefined	Undefined	Forbidden	Undefined	Forbidden	Forbidden
Belgium	No restrictions	Yes	Yes	No	Allowed^(b)^	Allowed^(c)^	Allowed	Allowed^(d)^	Allowed^(e)^	Allowed^(g)^
Bulgaria	Couples/single women	Yes	Yes	No	Allowed^(b)^	Allowed^(c)^	Allowed	Allowed	Forbidden	Allowed
Croatia	Couples/single women	Yes	Yes	No	Undefined	Allowed^(c)^	Allowed	Allowed^(d)^	Forbidden	Undefined
Cyprus	Married couples	Yes	UN	UN	Undefined	Undefined	Undefined	Undefined	Forbidden	Undefined
Czech Republic	Married couples	Yes	No	No	Allowed^(b)^	Allowed^(c)^	Undefined	Undefined	Forbidden	Allowed
Denmark	Couples/single women	Yes	Yes	No	Allowed^(b)^	Allowed^(c)^	Allowed	Allowed^(d)^	Forbidden	Allowed^(g)^
Estonia	Couples/single women	Yes	Yes	No	Undefined	Undefined	Allowed	Allowed	Forbidden	Allowed
Finland	Couples/single women	Yes	Yes	No	Allowed^(b)^	Allowed^(c)^	Allowed	Allowed	Forbidden	Allowed^(g)^
France	Married or stable couples	Yes	Yes^(a)^	No	Allowed^(b)^	Allowed^(c)^	Allowed	Allowed	Forbidden	Allowed
Germany	Married or stable couples	Yes	Yes^(a)^	No	Allowed^(b)^	Allowed^(c)^	Allowed	Allowed	Forbidden	Forbidden
Greece	Women up to 50 years old	Yes	Yes	Yes	Allowed^(b)^	Allowed^(c)^	Allowed	Allowed^(d)^	Allowed^(e)^	Allowed^(g)^
Hungary	Couple/single women	Yes	Yes	No	Allowed^(b)^	Allowed^(c)^	Allowed	Allowed	Forbidden	Allowed^(g)^
Ireland	No specific legislation	UN	UN	UN	Undefined	Undefined	Allowed	Undefined	Forbidden	Forbidden
Italy	Married or stable couples	Yes	Yes	No	Forbidden	Forbidden	Forbidden	Allowed	Forbidden	Forbidden
Latvia	Couples/Single women	Yes	Yes	No	Undefined	Allowed^(c)^	Allowed	Allowed	Forbidden	Allowed
Lithuania	Undefined	UN	UN	UN	Undefined	Undefined	Undefined	Undefined	Forbidden	Undefined
Luxembourg	Undefined	UN	UN	UN	Undefined	Undefined	Undefined	Undefined	Forbidden	Undefined
Malta	Undefined	UN	UN	UN	Undefined	Undefined	Undefined	Undefined	Forbidden	Undefined
Netherlands	Undefined	UN	UN	UN	Undefined	Undefined	Undefined	Undefined	Forbidden	Undefined
Poland	Undefined	UN	UN	UN	Undefined	Undefined	Undefined	Undefined	Forbidden	Undefined
Portugal	Married or stable couples	Yes	No	No	Allowed^(b)^	Allowed^(c)^	Allowed	Allowed	Forbidden	Allowed
Romania	Married or stable couples	Yes	Yes^(a)^	No	Undefined	Undefined	Allowed	Allowed	Forbidden	Allowed
Slovakia	Married or stable couples	Yes	UN	UN	Undefined	Undefined	Undefined	Undefined	Forbidden	Undefined
Slovenia	Married or stable couples	Yes	UN	UN	Undefined	Undefined	Undefined	Allowed	Forbidden	Allowed
Spain	No restrictions	Yes	Yes	No	Allowed^(b)^	Allowed^(c)^	Allowed	Allowed	Allowed^(f)^	Allowed^(g)^
Sweden	Married or stable couples	Yes	Yes^(a)^	No	Allowed^(b)^	Allowed^(c)^	Allowed	Allowed^(d)^	Forbidden	Allowed^(g)^
UK	Couples/single women	Yes	Yes	Yes	Allowed	Allowed	Allowed	Allowed	Allowed	Allowed^(g)^

(a): with restrictions; (b): in order to avoid genetic disease; (c): in order to avoid sex-linked disease; (d): up to 5 years; (e): between 6 months and 2 years after the donor's death; (f): up to 12 months after the donor's death; (g): allowed on embryos up to 14 days; UN: undefined.
